# N-Terminal Pro-B-Type Natriuretic Peptide as a Biomarker for Loss of Muscle Mass in Prevalent Hemodialysis Patients

**DOI:** 10.1371/journal.pone.0166804

**Published:** 2016-11-21

**Authors:** Misa Ikeda, Hirokazu Honda, Keiko Takahashi, Kanji Shishido, Takanori Shibata

**Affiliations:** 1 Division of Nephrology, Department of Medicine, Showa University School of Medicine, Tokyo, Japan; 2 Division of Nephrology, Department of Medicine, Showa University Koto Toyosu Hospital, Tokyo, Japan; 3 Division of Dialysis, Kitami Higashiyama Clinic, Tokyo, Japan; 4 Department of Medicine, Kawasaki Clinic, Kawasaki, Japan; Hospital Universitario de la Princesa, SPAIN

## Abstract

Protein-energy wasting (PEW) is common in hemodialysis (HD) patients. A recent study demonstrated that a high level of N-terminal pro-B-type natriuretic peptide (NT-proBNP) may be associated with PEW in those patients. This prospective study aimed to assess the association of NT-proBNP with body composition and muscle loss. A cohort of prevalent HD patients (n = 238) was examined. Blood samples were obtained at baseline to measure high-sensitive C-reactive protein (hsCRP), interleukin-6 (IL-6), adiponectin and NT-proBNP. Nutritional status and changes in muscle mass were assessed by subjective global assessment, percentage creatinine generation rate (%CGR), creatinine index (CI) and lean body mass (LBM) estimated by dual-energy X-ray absorptiometry (DXA). The %CGR and CI were calculated five times for one year, and DXA was performed at baseline and one year later. Cardiac function was estimated by ultrasonography at baseline. NT-proBNP was significantly higher in HD patients with PEW. High NT-proBNP was associated with cardiac dysfunction, increased levels of hsCRP and IL-6, and serially decreased levels of the indexes for muscle mass. Multiple regression analysis adjusted with confounders showed that NT-proBNP was an independent predictor for decrease in LBM and serial lower levels of %CGR and CI. In conclusion, the present study demonstrated a novel association between NT-proBNP and muscle loss. NT-proBNP may be an independent biomarker for malnutrition in HD patients, especially in patients with muscles loss, regardless of chronic inflammation, cardiac dysfunction, or overhydration.

## Introduction

Protein-energy wasting (PEW) is common in patients who undergo hemodialysis (HD). HD patients with PEW often show decreased skeletal muscle [1], which is a phenomenon similar to that of loss of muscle mass (sarcopenia), resulting from the process of aging [2–4].

Recent studies have shown that a high level of N-terminal pro-B-type natriuretic peptide (NT-proBNP) is associated with PEW and higher mortality in patients under HD [5,6]. B-type natriuretic peptide (BNP) is produced in the cardiac ventricles in response to pressure and volume overload, and is a biomarker of cardiac failure and coronary artery disease in the general population [7]. BNP is synthesized as a proBNP; then, it is further cleaved into bioactive BNP and biologically inactive NT-proBNP on a one-to-one basis [7].

Several factors influence the level of NT-proBNP including age, sex, body water status, cardiovascular disease, nutritional status, and kidney function [6,8]. Clinical studies have assessed the associations between NT-proBNP and nutritional status in patients with cardiac disease. Patients with congestive heart failure (CHF) often show a high level of NT-proBNP. Levels of NT-proBNP are substantially increased in patients with severe muscle wasting and cachexia [9,10]. Levels of NT-proBNP in patients with chronic kidney disease, especially those under HD, are usually increased as a consequence of increasing secretion and decreasing renal clearance [6,7]. In particular, remarkably higher levels of NT-proBNP seem to be an independent risk factor for PEW in patients under HD [1,2,6]. Therefore, NT-proBNP may be an important factor for the worsening of nutritional status. However, associations of NT-proBNP with change in body composition and muscle mass, which are key factors for sarcopenia, have not been fully evaluated in HD patients. Therefore, the aim of this study was to assess whether NT-proBNP is associated with decrease of muscle mass in patients under HD, compared with biomarkers of PEW.

## Methods

### Study design, setting and participants

This study was performed as part of an ongoing prospective cohort study that recruited 267 prevalent HD patients who had been undergoing HD for ≥ 6 months, were of age ≥ 20 years, and were treated at Kawasaki Clinic during the period July 2007 to July 2008. The study was designed as a prospective observational study with one-year follow-up after baseline analysis. Exclusion criteria were: expected death within 6 months, malignancy, infection, acute vasculitis, liver disease, and heart failure. The study protocol was approved by the ethics committee of Showa University School of Medicine. All subjects gave informed consent in accordance with the requirements of the institutional committee on human research; written informed consent was obtained from all patients. The study was performed according to the 2004 revised Helsinki Declaration.

Medical treatments used to manage the condition of all patients were similar, and the medication for each patient was prescribed according to the K/DOQI guidelines [11]. The patients were managed similarly in terms of protocols for HD, including prescription of dialysis dose. Basically, all recruited patients underwent routine hemodialysis 3 times/week (3–4 h/session) using conventional bicarbonate dialysate and standard high-flux cellulose triacetate, polysulfone, or other dialysis membranes. The patients were instructed to maintain a daily protein intake >1.0–1.2 g/kg body weight. Dialysis dosage was estimated by single pool (sp) Kt/V.

During the follow-up period, 29 patients left the study due to death (n = 13), transfer to another hospital (n = 5), or because dual-energy X-ray absorptiometry (DXA) data were not obtained after 12 months (n = 11). Hence, 238 patients completed the study.

### Biochemical methods

Creatinine, urea, and albumin (bromocresol purple) (Alb) were measured using routine procedures. The immunonephelometric method was used to measure high-sensitive C-reactive protein (hsCRP). Levels of interleukin (IL)-6 were measured using the QuantiGlo Human IL-6 ELISA Kit (R&D Systems Inc., Minneapolis, MN); the sensitivity of this kit was 0.16 pg/mL, with an intra-coefficient of variation (CV) <4.8% and an inter-CV <7.8%. Serum adiponectin levels were measured using the Human Adiponectin ELISA Kit (CircuLex, Nagano, Japan); the sensitivity of this kit was 1.46 ng/mL, with an intra-CV <5.0% and an inter-CV <4.4%. NT-proBNP levels were measured by fully-automated electrochemiluminescence [12], with an intra-CV <2.8% and an inter-CV <2.2%.

### Nutritional status and body composition

Nutritional status was assessed by subjective global assessment (SGA) and body mass index (BMI). SGA was scored according to the following items: 1) medical history (variation in weight, changes in dietary intake, digestive symptoms, physical activity, relationship between disease and nutrient requirements); and 2) physical examination (amount of fat and muscle loss, edema of the legs, presence of ascites).

Body composition was estimated by DXA on the day after a dialysis day [13]. Muscle mass was estimated by percentage creatinine generation rate (%CGR) and creatinine index (CI) [14,15]. DXA was assessed at baseline and at the one-year follow-up to estimate change in total fat mass (TFM) and lean body mass (LBM). %CGR and CI were calculated to estimate change in muscle mass once every three months (five times for one year). %CGR was calculated by Shinzato’s formula [16], and CI was calculated by the following formula: [17]

%CGR  =  Cs  ×  [7056  ÷  A  +  ΔBW  ÷  IBW  ×  240  ÷  (72 –  Td)]

$$\begin{array}{l} A\;=\;3354\;+\;\left(7.8\;\times \;Td\;+\;411\right)\;\times \;In\;\left(Cr\;÷\;Cs\right)\;–\;1.5\;\times \;Td\;–\;1449\;÷\;\left[\left(0.019\;\times \;Td\;+\;0.999\right)\;\times \;In\;\left(Ce\;÷\;Cs\right)\;–\;\left(0.00367\;\times \;Td\;–\;0.0219\right)\right] \end{array}$$

Cr  =  [−81.622  ×  In  (Ce  ÷  Cs)  ÷  60  ×  Td  +  0.942]  ×  Ce

$$\begin{array}{l} Cs:\;creatinine\;concentration\;at\;predialysis\;\left(\frac{mg}{dL}\right)\;,\\ Ce:\;creatinine\;concentration\;at\;postdialysis\;\left(\frac{mg}{dL}\right),\\ Td:\;HD\;duration\;time\;\left(hour\right),\;\Delta BW:\;change\;in\;body\;weight\;between\;pre-\\ \;and\;postdialysis\;\left(kg\right),\;IBW:\;ideal\;body\;weight\;=\;0.9\;\times \;\left(height\;–\;100\right) \end{array}$$

CI =  16.21  +  1.12  ×  [1  if male;  0  if female]  −  0.06  ×  age  (years)  −  0.08  ×  single pool Kt/V urea  +  0.009 x Cr  (mmol/L).

### Cardiac function

Ultrasound cardiography (UCG) was performed on the non-dialysis day after a dialysis session at baseline to estimate left ventricular end-diastolic dimension, left atrial dimension, ejection fraction, left ventricular posterior wall thickness, left ventricular mass index.

### Hydration status

Lean body mass calculated by DXA is possibly influenced by hydration status; therefore, we estimated total body water at baseline, 3, 6, 9, and 12 months by using the Watson formula as follows: [18,19]

Males:   2.447−  (0.09156  ×  age)  +  (0.1074  ×  height) +  (0.3362  ×  postdialysis body weight)

Females:  − 2.097  +  (0.1069  ×  height)  +  (0.2466  ×  postdialysis body weight)

Extracellular water was estimated by the Peter formula as follows: [20]

$$\begin{array}{l} Males:\;-\;2.47\;\times \;0.842\;+\;8.76\;\times \;body\;surface\;area\\ Females:\;-\;1.96\;\times \;0.527\;+\;8.05\;\times \;body\;surface\;area\\ Body\;surface\;area:\;0.5378\;\times \;postdialysis\;body\;weight\;\times \;0.3964\;\times \;0height\;\times \;0.3964\;65 \end{array}$$

### Statistical analyses

Data are presented as mean ± standard deviation or median (range) unless otherwise noted, and P values <0.05 were considered to indicate statistical significance. Comparisons between two groups for normally distributed variables were performed using Student’s t-test, and the Wilcoxon rank sum test was used for non-normally distributed variables. For nominal variables, Fisher’s exact test was used for comparisons between two groups. Correlations were calculated by Spearman’s rank correlation coefficient (ρ) for non-parametric data. Multivariate analysis of variance (MANOVA) was performed to assess the association between muscle mass (CGR, CI, LBM) as dependent variables and interactions of log NT-proBNP with cardiac function (left ventricular end-diastolic dimension, left atrial dimension, ejection fraction, left ventricular posterior wall thickness, left ventricular mass index) or biomarkers of log hsCRP, log IL-6 or adiponectin as independent variables at baseline. Associations between variables of muscle mass (CGR, CI, LBM) and hydration status (total body water and extracellular water) estimated by more than 2 equal time points and NT-proBNP were assessed by repeated measurement analysis of a multivariate approach. Independent associations between one dependent variable and more than two independent variables were assessed using multiple linear regression analysis or logistic regression analysis.

Data were analyzed using JMP Pro version 12.0.1 (SAS Institute, Cary, NC), Stata/MP 13.1 (StataCorp, College Station, TX) and Prism version 5.0c (GraphPad Software, Inc., La Jolla, CA).

## Results

### Characteristics of patients at baseline

The patient characteristics at baseline are presented in Table 1. There were 238 patients (149 men (64%), age 64 ± 13 years) analyzed in this study. Forty-nine percent of patients had a history of CVD, and 31% had diabetes mellitus. At baseline, 17% of the study patients were moderately to severely malnourished, as determined by SGA. The mean BMI level was 21 ± 3.0 kg/m^2^; 12.5% of the patients were underweight (BMI<18.5), and 11.0% of the patients were overweight (BMI>25).

**Table 1:** Patient characteristics and laboratory data at baseline in all patients and patients grouped according to NT-proBNP tertiles.

	All patients	Higher NT-proBNP^a^ (n = 78)	Middle- lower NT-proBNP (n = 160)	P value^b^
Age (years)	64 ± 13 (31, 89)^c^	69 ± 10 (43, 89)^c^	61 ± 13 (31, 89)^c^	<0.0001
Gender (% of men)	64	62	63	0.84
Dry weight (kg)	54.5	51.4	56.1	0.0008
BMI (kg/m^2^)	21.5 ± 3.0	20.6 ± 3.0	21.9 ± 3.9	0.05
Diabetes mellitus (%)	31	34	30	0.49
CVD (%)	49	63	42	0.002
Dialysis vintage (months)	174 ± 116	181 ± 119	172 ± 116	0.57
Primary disease (n, (%))				0.14
CGN	127 (53)	42 (15)	60 (32)	
DMN	67 (28)	23 (12)	45 (19)	
Nephrosclerosis	28 (12)	8 (1)	20 (5)	
Unknown	34 (14)	0 (0)	4 (2)	
Kt/V	1.44 ± 0.21	1.45 ± 0.21	1.44 ± 0.21	0.73
nPCR (g/kg/day)	1.02 ± 0.2	1.01 ± 0.2	1.02 ± 0.2	0.80
Malnutrition (%)	17	30	11	0.0002
SBP/ DBP (mmHg)	147 ± 23 / 81 ± 13	148 ± 24 / 82 ± 13	146 ± 23 / 81 ± 13	0.38 / 0.76
Total body water (L)	30.7 ± 4.4	29.5 ± 4.6	31.8 ± 5.5	0.001
Extracellular water (L)	11.5	11.0	11.7	0.001
ECW / TBW	0.37 ± 0.02	0.38 ± 0.02	0.37 ± 0.02	0.049
Hemoglobin (g/dL)	10.2 ± 1.0	10.3 ± 1.1	10.2 ± 0.9	0.48
Creatinine (mg/dL)	11.7 ± 2.7	10.5 ± 2.1	12.5 ± 2.8	<0.0001
Albumin (g/dL)	3.8 ± 0.3	3.8 ± 0.3	3.8 ± 0.4	0.67
High-sensitive CRP (mg/dL)	0.09 (0.05, 10.39)	0.16 (0.05, 10.39)	0.08 (0.05, 2.57)	0.02
Interleukin-6 (pg/ mL)	3.91 (1.2, 49.43)	4.57 (1.21, 49.43)	3.82 (1.20, 21.88)	0.008
Adiponectin (μg/mL)	18.2 ± 12.2	20.1 ± 15.7	17.2 ± 10.0	0.07
NT-proBNP (pg/mL)	2,910 (465, 78,400)	10,200 (5,760, 78,400)	1,890 (465, 5,590)	<0.0001
Ejection fraction (%)	65 ± 6	62 ± 8	65 ± 5	0.002
LAD (mm)	36.0 ± 6.0	39.7 ± 5.7	35.0 ± 5.5	<0.0001
LVDd (mm)	44.3 ± 6.1	45.7 ± 6.1	43.7 ± 6.0	0.01
LVPWT (mm)	11.3 ± 1.7	11.9 ± 1.8	11.1 ± 1.5	0.0005
Left ventricular mass index	139.7 ± 45.5	162.3 ± 47.9	128.5 ± 39.2	<0.0001
Total fat mass (g)	11,697 ± 4592	10,245± 4,042	12,514 ± 5,034	0.001
Lean body mass (g)	36,193 ± 7710	34,789 ± 6,913	37,162 ± 8,158	0.047
Creatinine generation rate (%)	110.0 ± 24.9	102.4 ± 22.4	113.2 ± 25.1	0.0004
Creatinine index (mmol/kg/day)	22.4 ± 2.9	21.0 ± 2.2	23.1 ± 2.9	<0.0001

^a:^ higher (H) and M—L (middle—lower) tertiles of N-terminal pro-B-type natriuretic peptide (NT-proBNP),

^b:^ H versus M—L tertiles of NT-proBNP;

^c:^ mean ± SD (range),

CGN: chronic glomerulonephritis, DMN: diabetic nephropathy, PCKD: polycystic zkidney disease, nPCR: normalized protein catabolic rate, ECW / TBW: extracellular water / total body water, LAD: left arterial dimension, LVDd: left ventricular end-diastolic dimension; LVPWT left ventricular posterior wall thickness.

### Correlations among NT-proBNP, variables for nutrition and inflammation, body composition, and cardiac function at baseline

Correlations among NT-proBNP, variables for nutrition and inflammation, body composition, and hydration status are shown in S1 Table. NT-proBNP was positively correlated with hsCRP, IL-6, percentage extracellular water, ratio of extracellular water to total body water (extracellular water / total body water); and inversely correlated with TFM, LBM, %CGR, and CI (S1 Table). LBM, %CGR, and CI were each positively correlated with one another (S1 Table). Associations between NT-proBNP and LBM at baseline and at 12 months later, or changes in LBM over 12 months are shown in S1 Fig. Changes in LBM over 12 months were significantly decreased above the threshold of log NT-proBNP of 8.5 (about 5,000 pg/mL) (S1 Fig).

NT-proBNP was positively correlated with left ventricular end-diastolic dimension, left atrial dimension, left ventricular posterior wall thickness and left ventricular mass index, and negatively correlated with ejection fraction (S2 Table).

MANOVA was conducted to estimate associations among variables for muscle mass, factors associated with NT-proBNP, and log NT-proBNP at baseline. Interaction between factors for cardiac function, factors of inflammation, or adiponectin and NT-proBNP were not associated with CI or %CGR. However, interactions between left ventricular end-diastolic dimension or adiponectin and NT-proBNP were significantly associated with baseline LBM.

### Changes of variables for nutrition and inflammation, and body composition according to NT-proBNP tertiles

Patient characteristics according to the higher (NT-proBNP ≥≧ 5,760 pg/mL) or middle–lower NT-proBNP tertiles (5,590 pg/mL ≥ NT-proBNP) are listed in Table 1. Patients with higher NT-proBNP were older and showed lower dry weight and total body water and extracellular water, high prevalence of history of CVD events and malnutrition, decreased levels of creatinine, increased levels of IL-6 and hsCRP, and poorer cardiac function (Table 1). Total fat mass and LBM in patients with higher NT-proBNP were lower than in patients with middle to lower NT-proBNP tertiles. Creatinine index as well as %CGR were significantly decreased in patients with higher NT-proBNP (Table 1).

**Fig 1:**
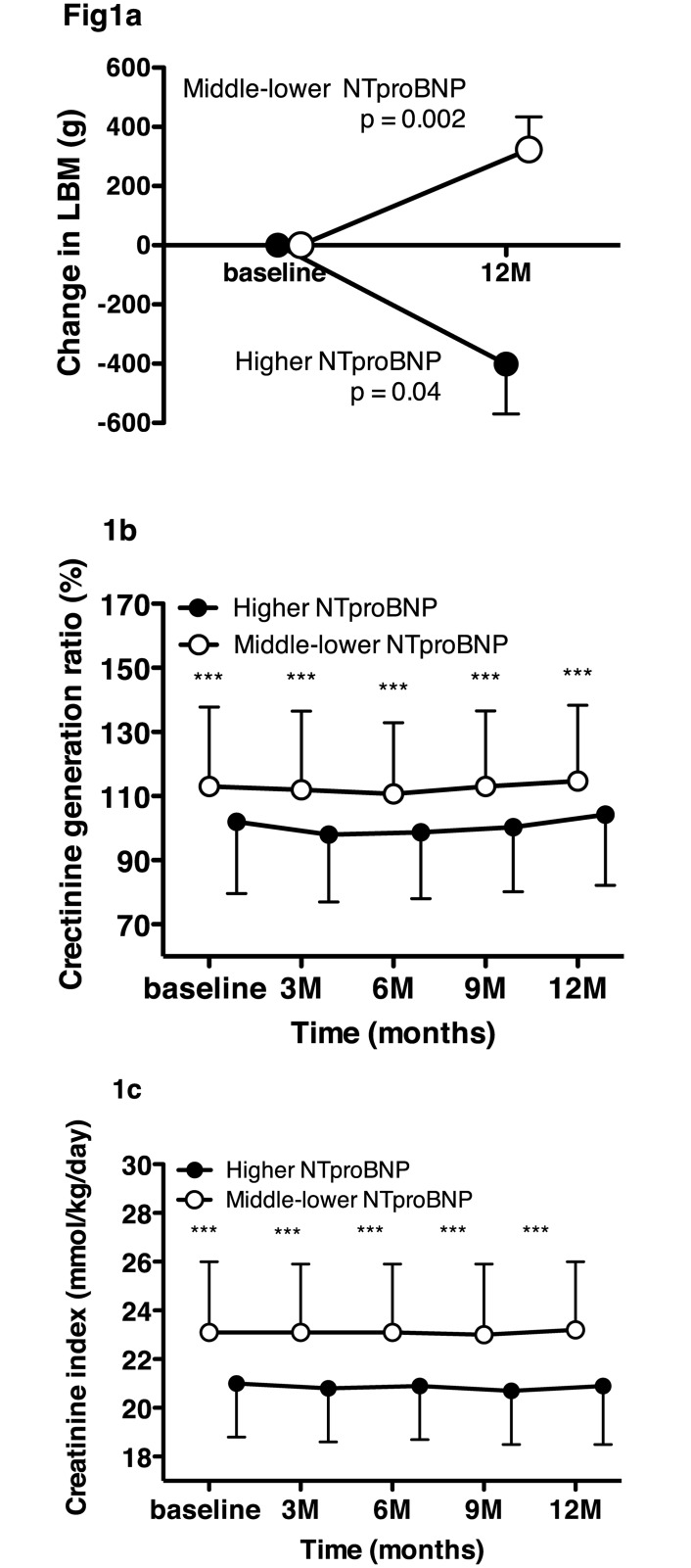
Changes in lean body mass over 12 months (mean ± SEM) between the patients in the higher and middle–lower tertiles of N-terminal pro-B-type natriuretic peptide (NT-proBNP) (a), changes in the percent creatinine generation rate between the patients in the higher and middle–lower tertiles of NT-proBNP (b), changes in creatinine index between the patients in the higher and middle–lower tertiles of NT-proBNP (c). *** P <0.001 between the higher versus middle–lower tertiles at the time point.

Values of LBM, but not of TFM, in the high tertile of NT-proBNP were significantly decreased during 12 months compared with the middle–lower NT-proBNP tertiles (Fig 1a). However, those changes were not obtained between the higher and lower–middle tertiles of hs-CRP, IL-6, and adiponectin. Changes of LBM in the lower–middle tertiles of NT-proBNP were increased as well as those of hs-CRP and IL-6 (S2 Fig).

The values of CI and %CGR in the higher NT-proBNP tertile were serially and continuously decreased during 12 months compared with the middle-lower NT-proBNP tertiles (Fig 1b and 1c). However, changes in those indexes did not differ between the higher tertile of hs-CRP, IL-6 or adiponectin and the middle-lower tertiles of hs-CRP, IL-6 or adiponectin, respectively (S3 and S4 Figs).

To estimate the influence of hydration status, changes in total body water and extracellular water as calculated by the Watson formula and the Peter formula, respectively, were assessed between the higher and the middle–lower NT-proBNP tertiles. The levels in total body water and extracellular water in the middle–lower NT-proBNP tertiles during 12 months were higher than those of the higher NT-proBNP tertile during 12 months (Table 1, S5 Fig).

Associations between NT-proBNP (higher tertile vs. middle–lower tertiles) and repeated measurement variables of muscle mass (%CGR, CI, LBM) and hydration status (total body water and extracellular water) were assessed by a multivariate approach. High NT-proBNP was significantly associated with levels of variables of muscle mass and hydration status (S3 Table). The interaction between NT-proBNP and time points for LBM was confirmed.

### Multivariate analysis for changes in LBM, %CGR, and CI

The association between NT-proBNP and changes in LBM was estimated by multivariate analysis (Table 2). NT-proBNP as well as hsCRP were significantly correlated with decreased changes in LBM for 12 months (Table 2, S4 Table). However, change in TFM did not correlate with NT-proBNP (data not shown).

**Table 2:** Multivariate analysis for changes of muscles.

Dependent factor: change in lean body mass (g/year)												
	Model 1			Model 2			Model 3			Model 4		
	B	SE	P	β	SE	p	β	SE	p	β	SE	p
log NT-proBNP	-366.0	94.6	0.0001	-352.5	94.4	0.0002	-371.5	97.4	0.0002	-406.0	107.7	0.0002
Dependent factor: muscles loss defined by % creatinine generation rate^a^												
	Model 5			Model 6			Model 7			Model 8		
	B	SE	P	β	SE	p	β	SE	p	β	SE	p
log NT-proBNP	0.38	0.15	0.01	0.39	0.16	0.01	0.35	0.16	0.02	0.45	0.17	0.008
Dependent factor: muscles loss defined by creatinine index^b^												
	Model 9			Model 10			Model 11			Model 12		
	B	SE	P	β	SE	p	β	SE	p	β	SE	p
log NT-proBNP	0.61	0.19	0.001	0.60	0.20	0.002	0.62	0.20	0.002	0.62	0.25	0.01

^a:^ muscles loss were defined as follow; the levels of %CGR would be changed down to < 100 or continuously decreased < 100 for 12 months.

^b:^ muscles loss were defined as follow; the levels of CI would be changed down to threshold of the lower tertile or continuously decreased in the lower tertile for 12 months.

Model 1, 5 and 9 include age, gender, diabetes mellitus status, past history of CVD, malnutrition estimated by subjective global assessment (SGA), HD vintage as independent factors.

Model 2, 6 and 10 include age, gender, diabetes mellitus status, past history of CVD, malnutrition estimated by SGA, HD vintage and log hs-CRP, log adiponectin and log NT-proBNP as independent factors.

Model 3, 7 and 11 include age, gender, diabetes mellitus status, past history of CVD, malnutrition estimated by SGA, HD vintage, and log IL-6, log adiponectin and log NT-proBNP as independent factors.

Model 4 includes age, gender, diabetes mellitus status, past history of CVD, malnutrition estimated by SGA, BMI, HD vintage, Kt/V, normalized protein catabolic rate, left ventricular end-diastolic dimension, left ventricular posterior wall thickness, left ventricular mass index, extracellular water / total body water, albumin, log hs-CRP, and log NT-proBNP as independent factors.

Model 8 includes age, gender, diabetes mellitus status, past history of CVD, malnutrition estimated by SGA, body mass index, HD vintage, Kt/V, normalized protein catabolic rate, left ventricular end-diastolic dimension, left ventricular posterior wall thickness, left ventricular mass index, extracellular water / total body water, albumin, and log NT-proBNP as independent factors.

Model 12 includes age, gender, diabetes mellitus status, past history of CVD, malnutrition estimated by SGA, body mass index, HD vintage, Kt/V, normalized protein catabolic rate, left ventricular end-diastolic dimension, left ventricular posterior wall thickness, left ventricular mass index, extracellular water / total body water, albumin, log adiponectin and log NT-proBNP as independent factors.

In the model 4, 8 and 12, the biomarker (hs-CRP, IL-6 or adiponectin) was selected as an independent factor according to findings of model 2, 3, 6, 7, 10 and 11 in S4 Table.

The association between NT-proBNP and changes in %CGR and CI is shown in Table 2. Multivariate models showed that NT-proBNP levels were significantly associated with muscle loss defined by %CGR or CI; if the levels of %CGR were to be changed down to < 100 or continuously decreased to < 100 for 12 months (n = 83, 36%), and the levels of CI were to be changed down to the threshold of the lower tertile or continuously decreased in the lower tertile for 12 months (n = 78, 33%), the patient was identified as having muscle loss. The associations between hsCRP, IL-6 or adiponectin and changes in %CGR and CI are shown in S4 Table.

## Discussion

The present study demonstrated the association of NT-proBNP with muscle loss in malnourished HD patients, compared with hsCRP, IL-6 and adiponectin, which are biomarkers of PEW [21].

NT-proBNP is a biomarker for cardiac stress and fluid overload [7]; therefore, NT-proBNP levels are influenced by hydration status in HD patients. Indeed, NT-proBNP levels were associated with factors of cardiac function in the present study, while NT-proBNP levels were significantly higher in HD patients with muscle loss. However, NT-proBNP independently predicted the decreased change of LBM and the index of muscle loss in the multivariate model adjusted with confounders including cardiac function. Patients with chronic heart failure tend to show high NT-proBNP levels; however, the levels are higher in patients with cachexia than in patients without cachexia [22]. Booth et al. reported the relationships among cardiac function, hydration status as estimated by bio-impedance, and NT-proBNP levels in HD patients [23]. This study showed that NT-proBNP levels are influenced by hydration status rather than cardiac dysfunction, and that hydration status as estimated by bio-impedance may be enhanced by malnutrition with loss of cell mass [23].

Madsen et al. reported a predictive ability of high NT-proBNP for mortality in patients under HD [24]. Interestingly, higher NT-proBNP levels, at both pre- and postdialysis, were significantly associated with increased mortality. Moreover, the predictive abilities were independent of volume overload, whereas NT-proBNP levels in patients under HD were elevated according to hydration status as well as reduced left ventricular ejection fraction and left ventricular hypertrophy [24].

NT-proBNP levels are often increased in patients with CHF and in patients under dialysis with concomitant inflammatory status [25]. Inflammation is a key factor for PEW in patients with end-stage kidney disease who are under dialysis [26]. Moreover, several clinical studies have demonstrated cross-talk between cardiac cachexia, BNP, and adiponectin in chronic heart failure patients [27,28]; this suggests that NT-proBNP and inflammatory biomarkers, and adiponectin interact with nutritional status in those patients. In the present study, NT-proBNP was correlated with hsCRP and IL-6 levels, and hsCRP predicted change in and loss of LBM. However, interactions of these factors with LBM, %CGR, and CI were not confirmed. Interaction between adiponectin and NT-proBNP was significantly associated with baseline LBM; however, the multivariate model showed that NT-proBNP was an independent predictor for muscle loss.

In the present study, serum albumin level was not associated with muscle loss. The serum albumin levels between patients with higher and middle to lower tertiles of NT-proBNP were similar, although the patients in the higher tertile of NT-proBNP showed a high prevalence of malnutrition compared with the patients in the middle to lower tertiles of NT-proBNP. Hypoalbuminemia often develops in patients with heart failure, resulting mainly from inflammation and cachexia [29]. On the other hand, albumin is not a reliable biomarker for malnutrition in patients under HD. The reason is that serum albumin levels are possibly influenced by protein loss through renal excretion and dialysis treatment, and by inadequate synthesis and catabolism resulting from insufficient diet and the unique situation in those patients, who experience such conditions as inflammation and chronic metabolic acidosis [30–33]. Thus, serum albumin cannot always be associated with malnutrition in patients under HD, although hypoalbuminemia is a risk for mortality in those patients [30]. A recent study demonstrated that low serum albumin levels were not associated with decreased muscle mass, but rather, with low muscle strength, in patients under HD [34]. Altogether, measurement of NT-proBNP, compared with serum albumin estimation, may be superior for identifying cardiac cachexia as well as muscle loss in patients under HD.

Overall, NT-proBNP may be an independent biomarker for muscle loss. Several hypotheses have been proposed concerning the association between high NT-proBNP and muscle loss. Natriuretic peptides (NPs) increase lipolysis in adipose tissue and mitochondrial oxidative capacity in human skeletal muscle, which prompts oxidative gene expression [35–38]. BNP could contribute to exercise-induced mitochondrial biogenesis in skeletal muscle [37], and is produced by activated satellite cells within ischemic skeletal muscle or by cardiomyocytes in response to blood pressure load, which regulates the regeneration of neighboring endothelia via receptor guanylyl cyclase-A [38]. However, BNP exerts strong lipolytic effects in humans [39]. A state of chronic heart failure causes increased NP production in the cardiac walls. NPs secreted into the circulation could stimulate the release of free fatty acids (FFAs) from adipose tissue. Adequate release of FFAs from adipose tissue is beneficial for cardiac metabolism; however, excessive and continuous release of FFAs may impair insulin sensitivity and cause myocardial lipotoxicity. Consequently, cardiac dysfunction may occur [39]. Moreover, levels of NPs in the circulation may produce increased energy dissipation by skeletal muscle [39] as well as adipose tissue [40,41]. Altogether, NT-proBNP may not only be an indirect biomarker for PEW due to heart failure and overhydration, but also for BNP-provoked loss of muscle mass in HD patients. Measurement of NT-proBNP levels could be a useful method for identifying dialysis patients at high risk for muscle mass loss.

However, the present study results must be considered with the following caveats. The number of patients was relatively small, and we could only evaluate the findings for prevalent HD patients. Additionally, the single-point measurement of NT-proBNP, as well as the other biomarkers of hsCRP, IL-6 and adiponection, did not permit accurate evaluation. Fahim et al. reported on the within–person coefficient of variation of NT-proBNP in patients under HD [42]. In the Fahim study, weekly and monthly variations of NT-proBNP were 27% and 35%, respectively; however, the values of NT-proBNP in patients with higher NT-proBNP were maintained at higher levels during the monitoring period [42]. Moreover, the variations of NT-proBNP were not associated with hydration status, inflammation, or cardiac comorbidity [42]. Thus, higher NT-proBNP levels in patients under HD are possibly continued at higher levels. Another limitation of the present study was that it excluded the patients who did not complete the analysis and who died during the one-year follow-up; thus, there may be a bias related to patients withdrawing from the study. Finally, we could not assess a specific association between LBM by DXA and hydration status estimated by an anthropometric method such as bio-impedance measurement. A large prospective cohort study is required to clarify the reliability of NT-proBNP as a predictor for progression of muscle loss in prevalent HD patients.

In conclusion, NT-proBNP may be an independent biomarker for PEW, especially in HD patients with muscle loss, regardless of chronic inflammation, cardiac dysfunction, or hydration status.

## Supporting Information

S1 Fig Associations between log N-terminal pro-B-type natriuretic peptide and lean body mass (LBM) at baseline (a), LBM at 12 months (b) or changes in LBM over 12 months (c). (TIFF)

S2 Fig Changes in lean body mass over 12 months (mean ± SEM) between the patients in the higher and middle–lower tertiles of high-sensitive CRP (hsCRP) (a), interleukin-6 (IL-6) (b), or adiponectin (c). (TIFF)

S3 Fig Changes in creatinine generation rate between the patients in the higher and middle–lower tertiles of high-sensitive CRP (hsCRP) (a), interleukin-6 (IL-6) (b), or adiponectin (c). (TIFF)

S4 Fig Changes in creatinine index between the patients in the higher and middle–lower tertiles of high-sensitive CRP (hsCRP) (a), interleukin-6 (IL-6) (b), or adiponectin (c). * P <0.05 between the higher versus middle to lower tertiles at the time point, respectively.
(TIFF)

S5 Fig Changes in total body water (TBW) (a), extracellular water (ECW) (b) and ratio of ECW to TBW (c) over 12 months between the patients in the higher and middle–lower tertiles of N-terminal pro-B-type natriuretic peptide (NT-proBNP). *** and ** P <0.001 and P <0.01 between the higher versus middle to lower tertiles at the time point, respectively.
(TIFF)

S1 Table Spearman’s rank correlation analysis among the biomarkers, body composition, indexes of muscles and hydration status at baseline. (DOCX)

S2 Table Spearman’s rank correlation analysis between NT-proBNP and cardiac functions at baseline. (DOCX)

S3 Table Association between NT-proBNP and repeated measurement variables by a multivariate approach. (DOCX)

S4 Table Multivariate analysis of biomarkers for changes in lean body mass, indexes of muscles. (DOCX)
