# Supplementary figures and images for: N-Terminal Pro-B-Type Natriuretic Peptide as a Biomarker for Loss of Muscle Mass in Prevalent Hemodialysis Patients
Source: PLoS One. 2016 Nov 21;11(11):e0166804. doi: 10.1371/journal.pone.0166804 (PMC5117720; doi:10.1371/journal.pone.0166804)

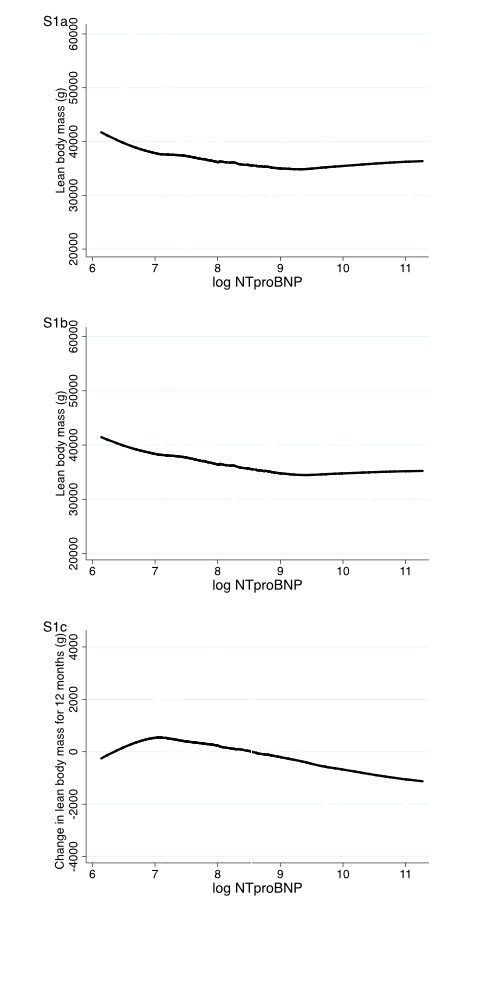

Supplement: S1 Fig — (TIFF) [file pone.0166804.s001.tiff]

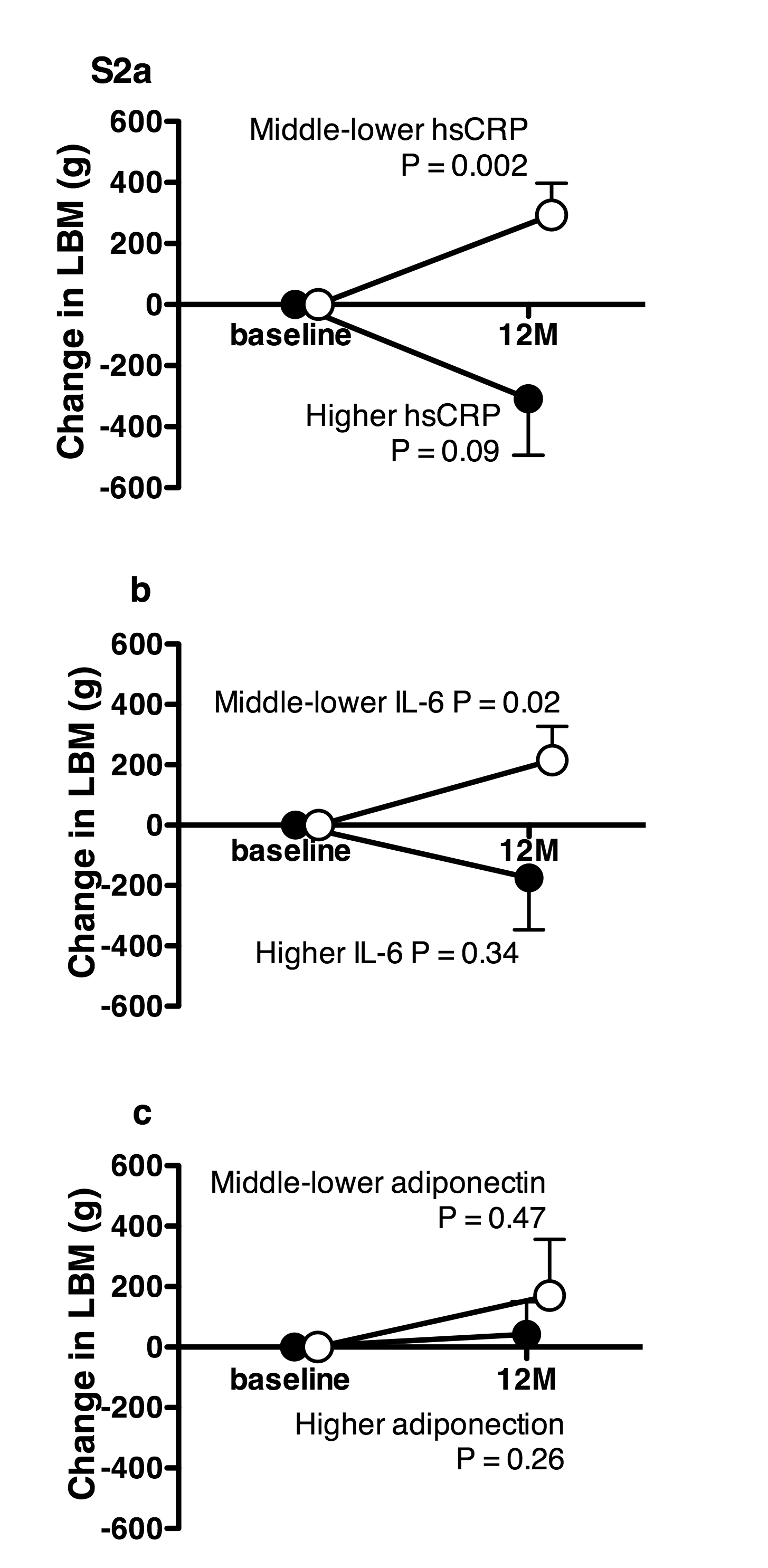

Supplement: S2 Fig — (TIFF) [file pone.0166804.s002.tiff]

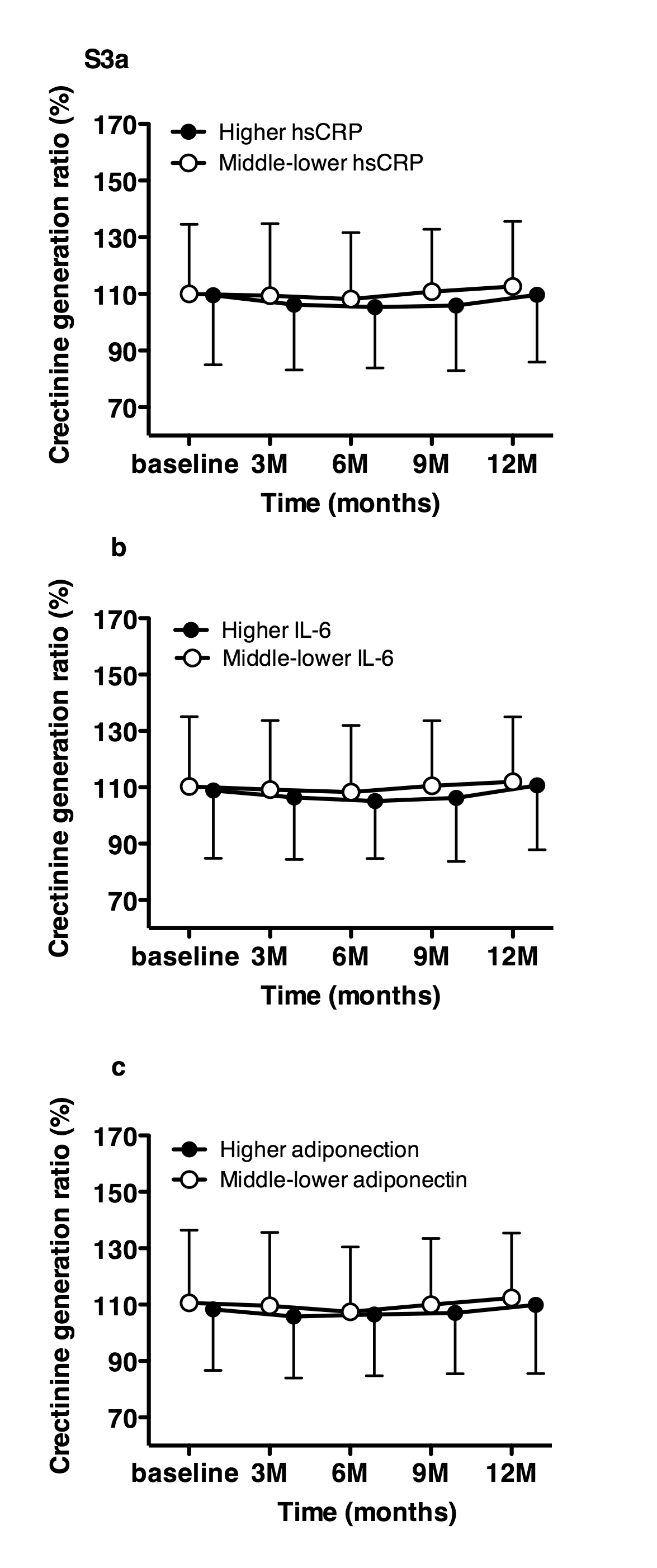

Supplement: S3 Fig — (TIFF) [file pone.0166804.s003.tiff]

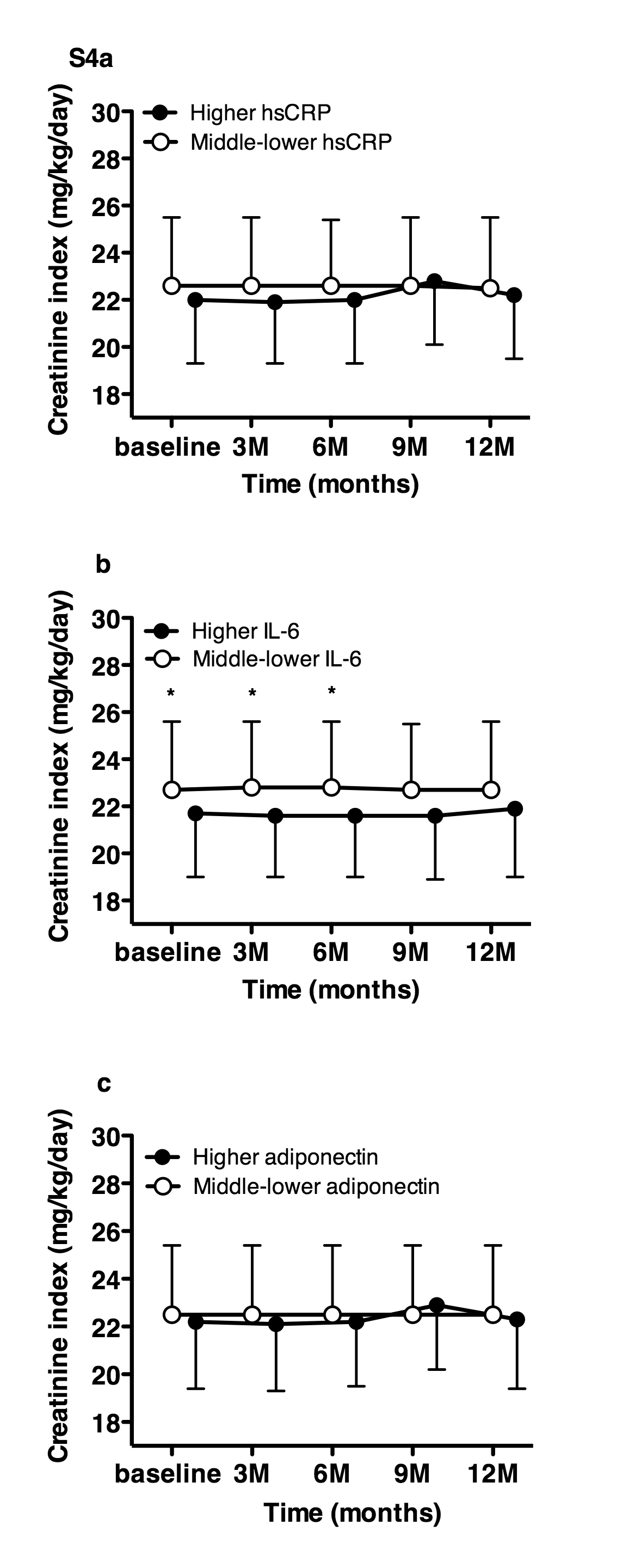

Supplement: S4 Fig — * P <0.05 between the higher versus middle to lower tertiles at the time point, respectively. (TIFF) [file pone.0166804.s004.tiff]

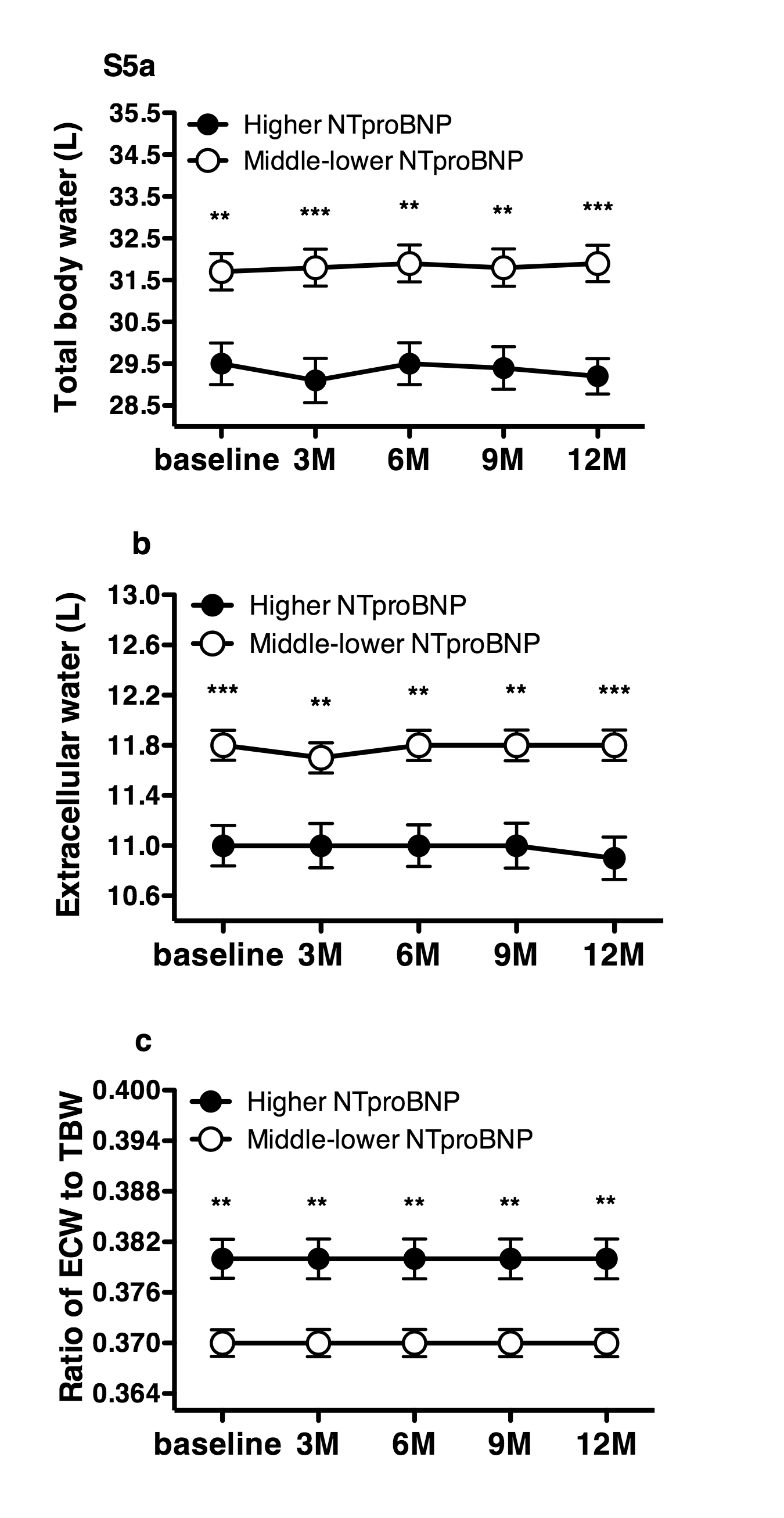

Supplement: S5 Fig — *** and ** P <0.001 and P <0.01 between the higher versus middle to lower tertiles at the time point, respectively. (TIFF) [file pone.0166804.s005.tiff]
