# Supplementary material for: N-Terminal Pro-B-Type Natriuretic Peptide as a Biomarker for Loss of Muscle Mass in Prevalent Hemodialysis Patients
Source: PLoS One. 2016 Nov 21;11(11):e0166804. doi: 10.1371/journal.pone.0166804 (PMC5117720; doi:10.1371/journal.pone.0166804)
Supplement: S1 Table — (DOCX) [file pone.0166804.s006.docx]

S1 Table. Spearman’s rank correlation analysis among the biomarkers, body composition, indexes of muscles and hydration status at baseline

|  | **LBM** | **%CGR** | **CI** | **Albumin** | **HsCRP** | **IL-6** | **ADN** | **NT-proBNP** | **%ECW** | **%TBW** | **ECW/TBW** |
| --- | --- | --- | --- | --- | --- | --- | --- | --- | --- | --- | --- |
| **TFM** | 0.02 | -0.001 | -0.003 | -0.01 | 0.19** | 0.11 | -0.18* | -0.19** | -0.73**** | -0.69**** | 0.23*** |
| **LBM** |  | 0.13* | 0.63**** | 0.08 | 0.08 | 0.02 | -0.18* | -0.16* | -0.64**** | 0.05 | -0.64**** |
| **%CGR** |  |  | 0.58**** | -0.06 | -0.05 | -0.16* | -0.10 | -0.14** | -0.03 | 0.02 | -0.05 |
| **CI** |  |  |  | 0.03 | -0.09 | -0.25*** | -0.16* | -0.38**** | -0.33**** | 0.28*** | -0.64**** |
| **Albumin** |  |  |  |  | 0.10 | 0.04 | -0.05 | 0.03 | -0.06 | 0.03 | -0.05 |
| **HsCRP** |  |  |  |  |  | 0.67**** | -0.05 | 0.16* | -0.22*** | -0.17** | 0.001 |
| **IL-6** |  |  |  |  |  |  | -0.06 | 0.27**** | -0.14* | -0.16* | 0.07 |
| **ADN** |  |  |  |  |  |  |  | 0.12 | 0.20** | 0.09 | 0.012 |
| **NT-proBNP** |  |  |  |  |  |  |  |  | 0.21** | 0.09 | 0.17** |
| **%ECW** |  |  |  |  |  |  |  |  |  | 0.63**** | 0.14* |
| **%TBW** |  |  |  |  |  |  |  |  |  |  | -0.68**** |

*: p<0.05, **: p<0.01, ***: p<0.001, ****: p<0.0001

TFM: total fat mass, LBM: lean body mass, %CGR: percentage creatinine generation rate, CI: creatinine index, ADN: adiponectin; %ECW: (extracellular water / body weight post HD) × 100, %TBW: (total body water / body weight post HD) × 100, ECW/TBW: extracellular water / total body water.
