# Supplementary material for: N-Terminal Pro-B-Type Natriuretic Peptide as a Biomarker for Loss of Muscle Mass in Prevalent Hemodialysis Patients
Source: PLoS One. 2016 Nov 21;11(11):e0166804. doi: 10.1371/journal.pone.0166804 (PMC5117720; doi:10.1371/journal.pone.0166804)
Supplement: S2 Table — (DOCX) [file pone.0166804.s007.docx]

S2 Table. Spearman’s rank correlation analysis between NT-proBNP and cardiac functions at baseline

|  | Left atrial dimension | Left ventricular end-diastolic dimension | LVPWT | Left ventricular mass index | Ejection fraction |
| --- | --- | --- | --- | --- | --- |
| NT-proBNP | 0.43*** | 0.13* | 0.30*** | 0.36*** | -0.18** |

LVPWT : left ventricular posterior wall thickness, *: p<0.05, **: p<0.01, ***: p<0.0001
