# Supplementary material for: N-Terminal Pro-B-Type Natriuretic Peptide as a Biomarker for Loss of Muscle Mass in Prevalent Hemodialysis Patients
Source: PLoS One. 2016 Nov 21;11(11):e0166804. doi: 10.1371/journal.pone.0166804 (PMC5117720; doi:10.1371/journal.pone.0166804)
Supplement: S3 Table — (DOCX) [file pone.0166804.s008.docx]

S3 Table. Association between NT-proBNP and repeated measurement variables by a multivariate approach

|  | LBM | | %CGR | | CI | | TBW | | ECW | |
| --- | --- | --- | --- | --- | --- | --- | --- | --- | --- | --- |
|  | Model 1 | | | | | | | | | |
|  | F | p value | F | p value | F | p value | F | p value | F | p value |
| Timepoint | 0.16 | 0.68 | 7.4 | <0.0001 | 2.1 | 0.07 | 2.4 | 0.04 | 4.9 | 0.0006 |
| NT-proBNP | 6.5 | 0.01 | 21.0 | <0.0001 | 41.1 | <0.0001 | 13.2 | 0.0004 | 13.1 | 0.0003 |
| Interaction of NT-proBNP and timepoint | 13.5 | 0.0003 | 0.9 | 0.45 | 0.5 | 0.69 | 2.1 | 0.06 | 4.2 | 0.002 |
|  | Model 2 | | | | | | | | | |
|  | F | p value | F | p value | F | p value | F | p value | F | p value |
| Timepoint | 0.20 | 0.65 | 7.4 | <0.0001 | 2.5 | 0.04 | 2.5 | 0.04 | 5.0 | 0.0005 |
| NT-proBNP | 6.7 | 0.01 | 14.0 | 0.0002 | 23.9 | <0.0001 | 20.9 | <0.0001 | 19.3 | <0.0001 |
| Interaction of NT-proBNP and timepoint | 13.1 | 0.0004 | 0.9 | 0.45 | 0.6 | 0.68 | 2.2 | 0.06 | 4.2 | 0.002 |

Model 1: Repeat measurement variables for lean body mass (LBM), percent creatinine generation rate (%CGR), creatinine index (CI), total body water (TBW) and extracellular water (ECW) were estimated with the timepoint of each repeat measurement variable, NT-proBNP (higher tertile vs. middle to lower tertiles), interaction of timepoint and NT-proBNP.

Model 2: Repeat measurement variables for LBM, %CGR and CI were estimated with age, sex, diabetes mellitus status, history of CVD, SGA, HD vintage, left ventricular end-diastolic dimension, left ventricular posterior wall thickness, ECW/TBW, the timepoint of each repeat measurement variable, NT-proBNP (higher tertile vs. middle to lower tertiles), interaction of timepoint and NT-proBNP. Repeat measurement variables for TBW and ECW were estimated with age, sex, diabetes mellitus status, history of CVD, malnutrition estimated by subjective global assessment, HD vintage, left ventricular end-diastolic dimension, left ventricular posterior wall thickness, the timepoint of each repeat measurement variable, NT-proBNP (higher tertile vs. middle to lower tertiles), interaction of timepoint and NT-proBNP.
