# Supplementary material for: N-Terminal Pro-B-Type Natriuretic Peptide as a Biomarker for Loss of Muscle Mass in Prevalent Hemodialysis Patients
Source: PLoS One. 2016 Nov 21;11(11):e0166804. doi: 10.1371/journal.pone.0166804 (PMC5117720; doi:10.1371/journal.pone.0166804)
Supplement: S4 Table — (DOCX) [file pone.0166804.s009.docx]

S4 Table. Multivariate analysis of biomarkers for changes in lean body mass, indexes of muscle

|  | Dependent factor: change in lean body mass (g/year) | | | | | | | | | | | |
| --- | --- | --- | --- | --- | --- | --- | --- | --- | --- | --- | --- | --- |
|  | Model 1 | | | Model 2 | | | Model 3 | | | Model 4 | | |
|  | Β | SE | P | Β | SE | P | β | SE | p | β | SE | P |
| log hs-CRP | **-283.4** | **82.4** | **0.0007** | **-323.6** | **80.8** | **0.004** | - | - | - | **-221.7** | **89.0** | **0.01** |
| log IL-6 | **-285.5** | **141.1** | **0.04** | - | - | - | -147.6 | 140.98 | 0.29 | - | - | - |
| log ADN | 272.3 | 180.7 | 0.13 | **351.2** | **173.8** | **0.04** | **357.6** | **177.3** | **0.04** | 315.5 | 180.3 | 0.08 |
|  |  | Dependent factor: muscles loss defined by % creatinine generation rate^a^ | | | | | | | | | |  |
|  | Model 5 | | | Model 6 | | | Model 7 | | | Model 8 | | |
|  | Β | SE | P | Β | SE | P | β | SE | p | β | SE | P |
| log hs-CRP | -0.06 | 0.13 | 0.61 | -0.12 | 0.13 | 0.38 | - | - | - | - | - | - |
| log IL-6 | 0.17 | 0.21 | 0.44 | - | - | - | 0.07 | 0.23 | 0.76 | - | - | - |
| log AND | 0.38 | 0.28 | 0.18 | 0.28 | 0.29 | 0.33 | 0.31 | 0.29 | 0.30 | - | - | - |
|  |  |  | Dependent factor: muscles loss defined by creatinine index^b^ | | | | | | | |  |  |
|  | Model 9 | | | Model 10 | | | Model 11 | | | Model 12 | | |
|  | Β | SE | P | Β | SE | P | β | SE | p | β | SE | P |
| log hs-CRP | -0.07 | 0.16 | 0.66 | -0.15 | 0.17 | 0.38 | - | - | - | - | - | - |
| log IL-6 | 0.05 | 0.27 | 0.83 | - | - | - | -0.23 | 0.29 | 0.34 | - | - | - |
| log AND | **1.06** | **0.35** | **0.003** | **0.93** | **0.37** | **0.01** | **0.93** | **0.37** | **0.01** | **1.24** | **0.41** | **0.003** |

a: muscles loss were defined as follow; the levels of %CGR would be changed down to < 100 or continuously decreased < 100 for 12 months.

b: muscles loss were defined as follow; the levels of CI would be changed down to threshold of the lower tertile or continuously decreased in the lower tertile for 12 months.

Model 1, 5 and 9 include age, gender, diabetes mellitus status, past history of CVD, malnutrition estimated by subjective global assessment (SGA), HD vintage and each variable.

Model 2, 6 and 10 include age, gender, diabetes mellitus status, past history of CVD, malnutrition estimated by SGA, HD vintage and log hs-CRP, log adiponectin and log NT-proBNP.

Model 3, 7 and 11 include age, gender, diabetes mellitus status, past history of CVD, malnutrition estimated by SGA, HD vintage, and log IL-6, log adiponectin and log NT-proBNP.

Model 4 includes age, gender, diabetes mellitus status, past history of CVD, malnutrition estimated by SGA, body mass index, HD vintage, Kt/V, normalized protein catabolic rate, left ventricular end-diastolic dimension, left ventricular posterior wall thickness, left ventricular mass index, extracellular water / total body water, albumin, log hs-CRP, and log NT-proBNP.

Model 8 includes age, gender, diabetes mellitus status, past history of CVD, malnutrition estimated by SGA, body mass index, HD vintage, Kt/V, normalized protein catabolic rate, left ventricular end-diastolic dimension, left ventricular posterior wall thickness, left ventricular mass index, extracellular water / total body water, albumin, and log NT-proBNP.

Model 12 includes age, gender, diabetes mellitus status, past history of CVD, malnutrition estimated by SGA, body mass index, HD vintage, Kt/V, normalized protein catabolic rate, left ventricular end-diastolic dimension, left ventricular posterior wall thickness, left ventricular mass index, extracellular water / total body water, albumin, log adiponectin and log NT-proBNP.
